# Do Judgments of Learning Impair Recall When Uninformative Cues Are Salient?

**DOI:** 10.3390/jintelligence11100203

**Published:** 2023-10-23

**Authors:** Kit S. Double

**Affiliations:** School of Psychology, The University of Sydney, Sydney, NSW 2006, Australia; kit.double@sydney.edu.au

**Keywords:** judgments of learning, reactivity, metacognition, memory, self-report, JOL

## Abstract

Judgments of learning (JOL) are one of the most commonly used measures of metamemory. There is mixed evidence that eliciting JOLs while participants are studying word pairs influences their subsequent recall, a phenomenon known as reactivity. The majority of studies have found that positive reactivity occurs when word pairs are related. This finding suggests that when the final test is sensitive to the cues used to make a JOL (e.g., pair relatedness), a benefit to recall is observed. Here, in three experiments, JOL reactivity is examined in the presence of a salient, yet non-diagnostic cue—font size. The results show that when study time is limited and font size is made salient, eliciting JOLs impairs future recall. It is argued that JOLs prompt participants to evaluate salient cues in the learning environment to evaluate whether they will affect future recall. This increased processing of salient cues can impair recall if it comes at the expense of processing less salient but more informative cues. These findings suggest that the relevance to the test of the cues processed when JOLs are performed determines the direction of reactivity effects, with both positive and negative reactivity being possible depending on how diagnostic the salient metacognitive cues are for recall.

## 1. Introduction

Often, researchers interested in metacognitive processes have elicited judgments of learning (JOL) as a means for measuring metamemory. JOLs require participants to self-assess the likelihood that they will recall the encoded material in the future. For example, how likely are you to remember that Bismarck is the capital of North Dakota if tested tomorrow? JOLs have provided substantial insights into the metacognitive beliefs that guide study behaviour and the metacognitive illusions to which we are sometimes susceptible. Traditionally it was assumed that eliciting JOLs from participants was an unobtrusive procedure that did not influence the underlying memory process. However, there have been several studies that have provided evidence that JOLs can sometimes be *reactive*, that is, they either impair or facilitate future recall (Ariel et al. 2021; Double et al. 2018; Halamish and Undorf 2023; Janes et al. 2018; Maxwell and Huff 2022, 2023; Mitchum et al. 2016; Rhodes and Tauber 2011; Rivers et al. 2021; Shi et al. 2023; Soderstrom et al. 2015). Most studies have focused on the influence of pair relatedness on reactivity, with evidence suggesting that reactivity occurs because JOLs facilitate the processing of relatedness cues which improve later recall (Janes et al. 2018; Rivers et al. 2021). Here, these findings are extended by examining reactivity when JOLs are based on a salient but uninformative cue—font size.

## 2. JOL Reactivity Research

A wide range of metacognitive measures have been found to be reactive including confidence ratings (Double and Birney 2017a, 2017b, 2018, 2019a; Lei et al. 2020), making predictions regarding whether stimuli will be remembered or forgotten (Murphy et al. 2023), think-aloud protocols (Fox and Charness 2010), and perhaps most frequently, judgments of learning (Double et al. 2018; Halamish and Undorf 2023; Janes et al. 2018; Maxwell and Huff 2022, 2023; Mitchum et al. 2016; Rivers et al. 2021; Shi et al. 2023; Soderstrom et al. 2015). Research concerning JOL reactivity initially focused on comparing the effect of performing delayed versus immediate JOLs on recall. A meta-analysis by Rhodes and Tauber (2011) found a very small but reliable positive effect (*g* = 0.08) of eliciting delayed JOLs compared with immediate JOLs. Recently, attention has turned to whether immediate JOLs are reactive. The evidence for reactivity to immediate JOLs is mixed. Many studies have found evidence of positive reactivity (Dougherty et al. 2005; Halamish and Undorf 2023; Janes et al. 2018; Maxwell and Huff 2022; Shi et al. 2023; Soderstrom et al. 2015; Witherby and Tauber 2017; Yang et al. 2015; Zechmeister and Shaughnessy 1980), others have found no reactivity (Ariel et al. 2021; Dougherty et al. 2018; Kelemen and Weaver 1997; Tauber et al. 2015; Tauber and Rhodes 2012), while some studies have found negative reactivity (Mitchum et al. 2016; Zhao et al. 2023). A recent meta-analysis by Double et al. (2018) found that, overall, there is no significant effect of JOLs on recall; however, this effect was moderated by pair relatedness. They found that there was significant positive reactivity for related word pairs (*g* = 0.32), but no effect of JOLs on unrelated word pair recall.

## 3. Cue-Strengthening Hypothesis

There are several theories that have been proposed to account for reactivity (Double and Birney 2019b; Halamish and Undorf 2023; Mitchum et al. 2016; Soderstrom et al. 2015). Several of these theories rely on strategic changes made by participants in response to JOLs. For example, Mitchum et al. (2016) proposed that JOLs prompt participants to reallocate their study time such that they overstudy easier items and understudy more difficult items, resulting in negative reactivity. Other theories of reactivity link reactivity effect to changes in the attentional processing of the cues when making JOLs (Double and Birney 2019b; Halamish and Undorf 2023; Soderstrom et al. 2015).

Reactivity has often been explained by the strengthening of cues used during the JOL. Koriat’s (1997) dual-basis view of metacognitive judgments distinguishes between experience-based cues and theory-based cues. JOLs often rely on experience-based cues such as the ease of processing or retrieval fluency (Begg et al. 1989; Benjamin et al. 1998). When making a JOL, participants draw on information from whatever cues are salient (either experience-based cues or theory-based cues) to make their prediction (Koriat 1997). According to this account, positive reactivity occurs when there is a match between the cues utilised during the JOL and the cues present when tested. Soderstrom et al. (2015) argued that reactivity is only observed in related word pairs because JOLs utilise the relatedness of the pair as a cue. This relational processing then facilitates recall of the target when given the cue during the criterion test.

## 4. Cue-Processing Account of Reactivity

While the cue-strengthening hypothesis accounts for positive reactivity, it does not provide a mechanism to explain negative reactivity effects, which have been observed several times (Mitchum et al. 2016; Zhao et al. 2023). Cue-processing accounts of reactivity propose a broader view of reactivity effects, by proposing that the *magnitude* and *direction* of reactivity is determined by the quality of the cues used to make the metacognitive judgment (Double and Birney 2019b). According to these accounts, metacognitive ratings direct attention to the cues that are processed when making the metacognitive judgment. Thus, if the final test is sensitive to these same cues, the additional processing facilitates recall (as proposed by the cue-strengthening hypothesis). However, if JOLs encourage additional processing of irrelevant cues, particularly at the expense of processing relevant cues, then the cue-processing account proposes that negative reactivity should occur. These accounts are in line with the idea that individuals flexibly attend to and integrate information from multiple cues when making metacognitive judgments (Undorf and Bröder 2020; Undorf et al. 2018).

Cues that are likely to be processed when making a JOL will range from experience-based cues (e.g., word frequency, pair relatedness, font size) to theory-based cues (e.g., confidence in one’s ability, belief that recall is easy, etc.). Crucially, some of these cues will be processed when making the JOL but deemed irrelevant to future recall. For example, if memorising a series of words where the font colour keeps changing, a participant may considerer the effect of colour on recall and then decide it is not relevant. JOLs will therefore direct cognitive resources to the processing of salient cues that are *candidates* for impacting future recall.

However, if cues are not relevant to the test, they may even impair test performance. For instance, if resources are expended processing uninformative cues, such as perceptual characteristics, then future recall may be impaired because this processing may come at the expense of processing less salient but more informative cues. The cue processing account of reactivity therefore predicts that reactivity is determined by the extent to which salient cues in the learning environment are *informative* for performance on the criterion test, regardless of whether they ultimately affect the JOL itself. Based on this account, it is hypothesised that if JOLs are elicited when uninformative cues are salient, recall will be impaired.

## 5. Current Study

This study aims to show that JOL reactivity is determined by the informativeness of the cues used to make the JOL. Specifically, this study will be the first to examine reactivity to JOLs when an uninformative cue, font size, is made salient. Notably font size has been shown to have either no effect on recall (Rhodes and Castel 2008) or, at best, a small effect on recall (Chang and Brainerd 2022; Luna et al. 2018; Undorf and Zimdahl 2019). Thus, when we refer to font size as an uninformative cue, we regard it as uninformative in that it provides *relatively* little diagnostic information to facilitate test performance (compared to pair relatedness, for example), rather than to imply that font size does not affect recall under any circumstance.

Despite having little or no effect on recall, evidence suggests that font size affects JOLs (Chang and Brainerd 2022; Luna et al. 2018). There are several moderators of the effect of font size on JOLs (Chang and Brainerd 2022; Kornell et al. 2011) including individual differences in beliefs regarding the effect of font size on memory (Su et al. 2018). While font size may or may not influence JOLs in all circumstances, importantly, the cue processing account says that even when a cue is not ultimately utilised (i.e., a participant decides it is unlikely to affect their future recall), if it is salient, then it will most likely be processed (i.e., a participant considers the *potential* effect of font size on their future recall) to a greater extent by participants who make a JOL as they evaluate whether the cue will affect future recall.

Briefly, Experiments 1 and 2 examined JOL reactivity when an uninformative cue (i.e., font size) was made salient in related and unrelated word-pair lists, respectively. Experiment 3 repeated the design of Experiment 1 but allowed participants unlimited study time in order to see if negative reactivity was driven by the combination of uninformative salient cues and the limited availability of cognitive resources. If, as suggested by the cue processing account of reactivity, reactivity is determined by the match between salient cues in the learning environment and the cues to which the criterion test is sensitive, then we would not expect JOLs to benefit recall if uninformative cues are made salient. In addition, if JOLs prompt participants to spend more cognitive resources processing salient uninformative cues (i.e., font size) at the expense of processing less salient but more informative cues, then negative reactivity should be observed.

## 6. EXPERIMENT 1

In Experiment 1, participants memorized a list of related word pairs, either with or without providing JOLs. For half of the participants, word pairs were presented in a font-inconsistent fashion with half of the pairs in small font and half of the pairs in large font. In the consistent font condition, participants learnt the same list of related word pairs, but all word pairs were presented in the same size font.

### 6.1. Method

#### Participants

Participants were recruited from Amazon’s Mechanical Turk. Participation was restricted to US participants and those who had over a 90% approval on the site, as well as reported being a native English speaker. Sample size was determined based on a power analysis (ANOVA F-test, f = 0.2, α = 0.80, 2-sided). A total of 80 participants were randomly assigned to the JOL condition and 80 participants were assigned to the control condition who performed the task without providing JOLs (No JOL). Half of the participants in each condition performed the task with inconsistent font sizes, while the other half performed the task with all word pairs in a consistent font size. Two participants in the JOL condition did not answer any of the JOLs and their data were excluded. A total of 59.38% of participants were female and the mean age was 39.74 years (SD = 12.56 years).

### 6.2. Materials and Procedure

A total of 30 related word pairs (e.g., flu–sick) were generated using norms from the University of South Florida Free Association Norms (Nelson et al. 2004). The word pairs had a mean forward associative strength of 0.53 (range 0.43 to 0.69). Pairs are available on the Open Science Framework at https://osf.io/grv3u/. The experiment was coded in HTML/Java using the ‘Collector’ program (Garcia and Kornell 2014). An experimenter-paced study was utilised with participants studying the 30 word pairs for 8 s each (as with Soderstrom et al. 2017). Participants in the JOL condition were also required to make their JOL during this time. To equate exposure time the word pair remained on the screen for the full 8 s duration regardless of when participants made their JOL. The JOL was worded as “How likely are you to correctly recall this item on a later test?”. Participants were asked to enter a value between 0 and 100 using their keyboards. Word pairs were presented in a random order that was determined anew for each participant. In addition, for participants in the inconsistent font condition, half of the word pairs were presented in regular-size font, while half were presented in a large font. While the exact font size was determined by a participant’s computer hardware, the large font was always 4 times larger than the regular font (approximately 12 pt/48 pt). The font size was again randomly determined for each word pair and each participant anew, such that half of the word pairs were always displayed in regular font and half were always displayed in large font in an intermixed fashion. For participants in the consistent-font-size condition, all word pairs were presented in regular font size (approximately 12 pt). We opted to use this font size only (as opposed to allocating participants to 12/48 pt in a consistent manner) because it was thought to be “typical” in the minds of participants (in terms of what they were used to seeing on a computer screen). The concern with using consistent 48 pt font was that this would unintentionally draw attention to font size simply because it was not what participants were likely anticipating.

After a three-minute filler task of playing Tetris, participants were required to complete a recall test. The test presented each of the 30 cues and participants were required to input the target using their keyboards. Each of the 30 targets was displayed in regular font during the test so that the font size would remain as an uninformative cue. No feedback was provided during the test. The test was scored such that minor spelling mistakes and incorrect pluralisation were scored as being correct.

### 6.3. Results and Discussion

Analysis was performed using R version 3.4.3 (R Core Team 2017).

#### 6.3.1. Judgement Type and Font Consistency

**Figure 1:**
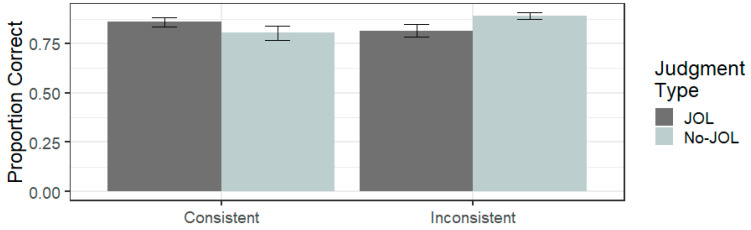
Mean proportion correctly recalled as a function of judgment type and font consistency in Experiment 1. Error bars represent ± 1 standard error.

To compare reactivity in the consistent-font-size condition with the inconsistent-font-size condition a 2 (judgment type: JOL vs. No JOL) × 2 (font consistency: inconsistent vs. consistent) between-subject ANOVA, collapsing across all font sizes, was performed next. The overall proportion of word pairs recalled on the test was 0.84 (SD = 0.18). The ANOVA indicated that there was no significant difference in recall between the consistent-font-size condition (*M* = 0.83, SD = 0.20) and the inconsistent-font-size condition (*M* = 0.85, SD = 0.16, *F*(1,156) = 0.563, η_p_^2^ = 0.004, *p* = 0.454). Similarly, there was no overall difference in recall performance between the JOL group (*M* = 0.84, SD = 0.18) and the No JOL group (*M* = 0.85, SD = 0.19, *F*(1,156) = 0.141, η_p_^2^ < 0.001, *p* = 0.708). Crucially, the judgment type × font consistency interaction was significant (*F*(1,78) = 5.198, η_p_^2^ = 0.032, *p* = 0.024), as shown in Figure 1. Follow-up planned pairwise comparisons were carried out to probe the interaction further. As hypothesised, the JOL group recalled significantly fewer words than the No JOL group with the inconsistent font condition (t(78) = 2.133, *p* = 0.037, *d* = −0.48). With the consistent font condition, there was no significant difference between the JOL condition and the No JOL condition (t(78) = 1.22, *p* = 0.228, *d* = 0.27).

#### 6.3.2. Font Size Effects on Recall and JOLs

As font size only varied in the inconsistent-font-size condition, the analysis of font size effects was confined to those participants in the inconsistent-font-size condition (n = 80). A 2 (judgment type: JOL vs. No JOL) × 2 (font size: large vs. regular) between–within-subject design analysis of variance (ANOVA) was performed on participants in the inconsistent-font-size condition. The ANOVA indicated that recall of large-font pairs (*M* = 0.86, SD = 0.17) was not significantly different to regular-font word pairs (*M* = 0.84, SD = 0.18, *F*(1,78) = 1.84, η_p_^2^ = 0.023, *p* = 0.179). Crucially, the JOL group (*M* = 0.81, SD = 0.21) had significantly poorer recall compared with the No JOL group (*M* = 0.89, SD = 0.12, *F*(1,78) = 4.55, η_p_^2^ = 0.055, *p* = 0.036). The judgment × font size interaction was not significant (*F*(1,78) = 3.101, η_p_^2^ = 0.038, *p* = 0.082). In addition, a comparison of group means indicated that there was no significant difference in JOLs for regular-font items (*M* = 64.00, SD = 27.80) compared with the large-font items (*M* = 63.70, SD = 26.59, t(39) = 0.252, *p* = 0.802, *d* = −0.04), suggesting that font size did not affect JOLs.

## 7. EXPERIMENT 2

The results of Experiment 1 suggested that when salient uninformative cues were present in the learning environment, performance on a later recall test was impaired for participants performing JOLs. These results are particularly notable given that the experiment utilised related word pairs where positive reactivity was typically observed (Double et al. 2018). Experiment 2 was designed to confirm and extend these findings to unrelated word pairs.

### 7.1. Method

#### Participants

Participants were recruited using the same procedure as for Experiment 1. As with Experiment 1, 160 participants were randomly allocated between subjects to a judgment condition and font size consistency in a balanced design, such that 40 participants were in each of the 4 conditions. A total of 19 participants failed to recall any of the word pairs correctly and 1 participant in the JOL group did not provide any JOLs, so their data were excluded from the analysis. An additional 20 participants were recruited as replacements using the same recruitment procedure. The final sample (64% female) had a mean age of 39.03 years (SD = 11.69).

### 7.2. Materials and Procedure

The procedure was the same as that described for Experiment 1, except that unrelated word pairs were used. A total of 30 unrelated word pairs were generated by randomly pairing words from the University of South Florida Free Association Norms (Nelson et al. 2004).

### 7.3. Results and Discussion

#### 7.3.1. Judgement Type and Font Consistency

The overall proportion of word pairs recalled on the test was 0.36 (SD = 0.29).

**Figure 2:**
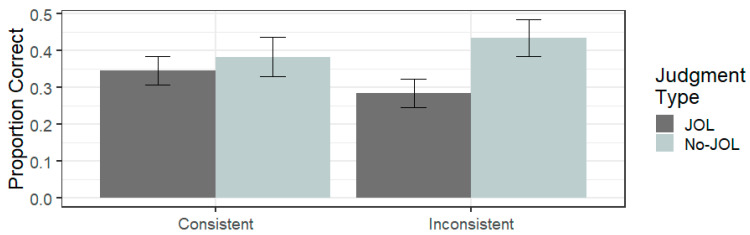
Mean proportion correctly recalled as a function of judgment type and font consistency in Experiment 2. Error bars represent ± 1 standard error.

A 2 (judgment type: JOL vs. No JOL) × 2 (font consistency: inconsistent vs. consistent) between-subject ANOVA, collapsing across all font sizes, was performed. As is depicted in Figure 2, the ANOVA indicated that there was no significant difference in recall between the consistent-font-size condition (*M* = 0.37, SD = 0.29) and the inconsistent-font-size condition (*M* = 0.36, SD = 0.29, *F*(1,156) = 0.015, η_p_^2^ < 0.001, *p* = 0.904). The ANOVA indicated that overall, recall performance was poorer in the JOL group (*M* = 0.32, SD = 0.25) compared to the No JOL group (*M* = 0.41, SD = 0.32, *F*(1,156) = 4.128, η_p_^2^ = 0.026, *p* = 0.044). The judgment type × font consistency interaction was not significant (*F*(1,156) = 1.521, η_p_2 = 0.010, *p* = 0.219). Given the theoretical relevance, the planned pairwise comparisons were carried out to examine reactivity within the consistent font size and inconsistent font size groups, separately. As with Experiment 1, the JOL group recalled significantly fewer words than the No JOL group in the inconsistent font condition (t(78) = 2.34, *p* = 0.022, *d* = −0.52). In the consistent font condition, again, there was no significant difference between the JOL condition and the No JOL condition (t(78) = 0.56, *p* = 0.579, *d* = −0.12). Based on the fact that the interaction term was not significant in Experiment 2, as opposed to Experiment 1, it appears to be driven by a negative reactivity effect, albeit a non-significant effect, in the consistent font condition. This has been observed in previous studies (Mitchum et al. 2016).

#### 7.3.2. Font Size Effects on Recall and JOLs

A 2 (judgment type: JOL vs. No JOL) × 2 (font size: large vs. regular) between–within-subject design ANOVA was performed on participants in the inconsistent-font-size condition (n = 80). The ANOVA indicated that recall of large-font pairs (*M* = 0.35, SD = 0.30) was not significantly different to regular-font word pairs (*M* = 0.37, SD = 0.30, *F*(1,78) = 2.056, η_p_^2^ = 0.026, *p* = 0.156). As with Experiment 1, the JOL group (*M* = 0.28, SD = 0.26) had significantly poorer recall compared with the No JOL group (*M* = 0.43, SD = 0.33, *F*(1,78) = 5.465, η_p_^2^ = 0.065, *p* = 0.022). The judgment × font size interaction was not significant (*F*(1,78) = 0.025, η_p_^2^ < 0.001, *p* = 0.874). In addition, a comparison of means indicated there was no significant difference in JOLs for regular size font items (*M* = 37.52, SD = 23.25) compared with the large-font items (*M* = 38.56, SD = 24.00, t(39) = 0.77, *p* = 0.444, *d* = 0.12), suggesting that font size did not affect JOLs.

## 8. EXPERIMENT 3

Experiments 1 and 2 (inconsistent-font-size condition) both showed some evidence of negative reactivity to JOLs. The goal of Experiment 3 was to examine whether this negative reactivity was due to the reallocation of cognitive resources when the study time was experimenter-paced. The cue processing account suggests that direct attention to salient cues occurs as their potential effects on recall are evaluated. Presumably, this will only have a negative effect on future recall if this processing comes at the expense of processing informative cues. Notably, recent evidence suggests that reactivity is less pronounced when study time is self-paced (Janes et al. 2018); thus, it may be that reactivity effects are largely driven by the reallocation of resources under time pressure. To test this hypothesis, reactivity to related word pairs when uninformative cues were present (in a similar fashion to Experiment 1) was examined, but participants were allowed to study the word pairs for as long as they wanted. Without the demands of experimenter-paced study time, it was hypothesised that negative reactivity would not occur, even though uninformative salient cues were present.

### 8.1. Participants

Participants were recruited using the same procedure as for Experiments 1 and 2. Only an inconsistent-font-size condition was administered, with 80 participants randomly allocated between subjects to a judgment condition (40 JOL; 40 No JOL). A total of 62% of participants were female with a mean age = 42.19 years, SD = 12.0 years.

### 8.2. Materials and Procedure

The same word pairs that were developed for Experiment 1 were used. Similarly, the procedure was the same as that described in Experiment 1, except that participants were able to study each word pair for as long as they deemed necessary before moving on to the next pair. In addition, all series of word pairs were presented with inconsistent fonts, with half of the words presented in large font and half in regular font, which was randomly determined for each participant anew.

### 8.3. Results and Discussion

#### 8.3.1. Recall

**Figure 3:**
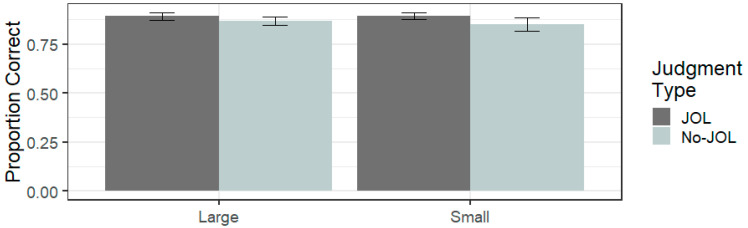
Mean proportion correctly recalled as a function of judgment type and font size in Experiment 3. Error bars represent ± 1 standard error.

The overall proportion of word pairs recalled on the test was 0.88 (SD = 0.13). Recall differences are presented in Figure 3. An ANOVA indicated there was no overall difference in recall performance between the JOL group (*M* = 0.89, SD = 0.11) and the No JOL group (*M* = 0.86, SD = 0.17, *F*(1,78) = 1.378, η_p_^2^ = 0.017, *p* = 0.244). Nor was there a significant difference between large (*M* = 0.88, SD = 0.12) and regular font sizes (*M* = 0.87, SD = 0.17, *F*(1,78) = 0.278, η_p_^2^ = 0.004, *p* = 0.600). Furthermore, the interaction between judgment type and font size was not significant (*F*(1,78) = 0.400, η_p_^2^ = 0.005, *p* = 0.529). These results suggest that, unlike under an experimenter-paced study time when participants can pace their own study time, negative reactivity is not observed in the presence of uninformative salient cues. This is consistent with other reactivity research, which has found little or no reactivity when study time is self-paced (Janes et al. 2018). However, it is also worth noting that performance was near the ceiling in this experiment and this may have limited our ability to observe reactivity effects.

#### 8.3.2. Study Time

A 2 (judgment type: JOL vs. No JOL) × 2 (font size: regular vs. large) between–within-subject ANOVA was performed on median study time. The ANOVA indicated that the JOL group (*M* = 6.59 s, SD = 3.96) spent longer studying each item (though this time also included the time to make the JOL) than the No JOL group (M = 2.98 s, SD = 2.34, *F*(1,78) = 25.23, η_p_^2^ = 0.244, *p* < 0.001). There was no significant difference in median response time between regular-font-size items (M = 4.77, SD = 3.73) and large-font-size items (M = 4.80, SD = 3.70, *F*(1,78) = 0.806, η_p_^2^ < 0.001, *p* = 0.802), nor was the interaction between font size and judgment type significant (*F*(1,78) = 0.50, η_p_2 = 0.006, *p* = 0.480).

#### 8.3.3. JOLs

There was a small significant difference in JOLs for regular-font items (*M* = 71.75, SD = 24.29) compared with the large-font items (*M* = 74.32, SD = 24.05, t(39) = 2.48, *p* = 0.018, d = 0.39), suggesting that participants were more confident recalling large-font items compared with regular-font items. It is difficult to explain exactly why we observed a significant effect of font size on JOLs observed here compared with the previous experiments. Although, this may suggest that font size may be utilised more readily when participants make JOLs with a self-paced study time compared with an experimenter-paced study time. The findings are, however, in keeping with a recent meta-analysis by Chang and Brainerd (2022) that found that the effect of JOLs on font size varies with study time. Of particular relevance, they found that an effect of font size on JOLs is only reliable for shorter study durations (2 and 5 s); notably, the effect size was much smaller (g = 0.13) when study times were 8 s, as was used in the previous experiment. Although speculative, this finding may be in keeping with the idea proposed by the cue-processing theory: participants consider the potential effect of font size on future recall; however, when given ample time, they ultimately decide against its potential effect. Given that the manipulation of font size is somewhat of a means to an end within the current design, we will reserve speculation and refer the interested reader to Chang and Brainerd (2022) for a discussion of the robustness and moderators of the font size illusion.

#### 8.3.4. Mini Meta-Analysis

In order to increase the reliability of the current findings a mini meta-analysis was also performed to combine the data from the three experiments. Mini meta-analyses are argued to improve the reliability and replicability of findings (Goh et al. 2016). Mini meta-analyses are particularly useful for detecting smaller effect sizes (Goh et al. 2016).

We meta-analysed our three experiments using random effects, in which the mean effect size (i.e., JOL vs No JOL) was weighted by sample size. The results of the meta-analysis suggested that there was no overall reactivity effect averaged across the three experiments (*g* = −0.08, *p* = 0.588). When font consistency was entered as a covariate, the results indicated that there was significant greater negative reactivity for inconsistent-font-size conditions (b = −0.50, *p* = 0.023); however, neither condition reached significance when considered on its own: inconsistent (*g* = −0.24, *p* = 0.33) versus consistent (*g* = 0.07, *p* = 0.712).

#### 8.3.5. General Discussion

This study was the first study to examine JOL reactivity when the presence of salient yet uninformative cues in the learning environment was manipulated. While previous studies have shown that JOLs can facilitate recall in related word pairs (Janes et al. 2018; Soderstrom et al. 2015; Witherby and Tauber 2017), the results here suggest that when study time is constrained, JOLs impair performance when there are salient uninformative cues in the learning environment. These results suggest that the magnitude and direction of reactivity to JOLs is at least, in part, determined by the salience and informativeness of cues in the environment and provides evidence for the cue processing account of reactivity. In addition, these findings raise further concerns for researchers who utilise JOLs to measure metacognition.

These results provide support for the central ideas of the cue processing account for reactivity, namely that JOLs prompt participants to process salient cues to decide whether or not to utilise them when making their JOL. This additional processing of salient cues may facilitate later recall if the cues being processed are beneficial to performance on the criterion test (e.g., the relatedness between a cue and target). However, when salient cues are uninformative (e.g., font size), additional processing of these cues may come at the expense of processing less salient but informative cues and thereby impair future recall performance. Furthermore, these results suggest that reactivity is determined not only by cues that are utilised in the JOL, but by cues salient in the learning environment, whether or not they are ultimately utilised when a participant makes a JOL.

There is an increasing body of research into reactivity and there remains ongoing debate regarding the mechanisms best able to explain reactivity effects. Several accounts of reactivity have emphasised the role of changes in attention and the role of cue processing (Double and Birney 2019b; Halamish and Undorf 2023; Soderstrom et al. 2015). The current findings seem to support this notion, suggesting that JOLs encourage a change in the processing of salient environmental cues. Evidence of reactivity is somewhat equivocal, with positive, negative, and no reactivity having been observed in the literature. While this could, of course, suggest that the effect is not robust, it seems likely that there are important moderators that theories of reactivity need to incorporate. These include both contextual variables, such as the cues salient in the learning environment, as well as individual differences (Birney et al. 2018; Double and Birney 2017a), and even the wording of the metacognitive rating itself (Double and Birney 2019a). However, while our findings are in keeping with the cue-processing theory, this interpretation relies on an indirect inference about the reallocation of attention when JOLs are elicited. The current experiments cannot directly test this assumption and further research is needed to try and directly observe any attentional effects of metacognitive ratings and whether these drive reactivity effects.

Arguably, reactivity is less of a threat to experimental validity if it occurs consistently across participants and stimuli characteristics (which often represent different experimental conditions). However, along with previous studies which suggest that reactivity exaggerates the effect of pair relatedness (e.g., Janes et al. 2018), the current results suggest that JOLs may interact with stimuli characteristics and possibly exaggerate the effect of stimuli characteristics and potentially between-condition differences. Consider, as an example, a relatively simple experimental design where a researcher modifies the font type between subjects and then examines the effect of font type on JOLs and recall. It is possible that any between-condition differences in recall are not a direct effect of the font type. Instead, they may be caused by the JOLs prompting participants to spend time processing the font type changes; therefore, differences in recall between font types may or may not appear when JOLs are not elicited. Halamish (2018) recently found evidence of this, showing that a very small font enhanced recall performance, but only when JOLs were not elicited. This result provides further evidence that JOLs might be an underrecognized moderator of the effect of stimuli characteristics on recall performance and it may be that researchers need to consider examining group differences with and without JOLs being elicited.

Notably, the present results suggest that reactivity may be affected by salient stimuli, regardless of whether or not they are ultimately used in the JOL itself. Therefore, researchers cannot rule out JOL reactivity simply because a stimuli characteristic does not produce a difference in the JOL. The cue-processing account of reactivity suggests that reactivity is driven by cues that are processed in a way that they would not otherwise have been in the absence of JOLs. I argue that JOLs prompt participants to evaluate the potential effect of salient cues on recall, and if processing these cues comes at the expense of informative but less salient cues, then negative reactivity is observed. However, if the salient cues are informative (i.e., related to performance on the criterion test), then positive reactivity may be observed.

The goal of this study was to examine whether reactivity effects depended on the salience of non-diagnostic cues. While we explored whether reactivity effects depended on the salience of non-diagnostic cues in different contexts (related word pairs and unrelated word pairs, and self- and experimenter-paced study), in order to test the generalizability of our conclusions, making comparisons across experiments should be carried out cautiously. Future research may wish to manipulate within-experiment factors such as relatedness and study pacing (along with other factors) in order to draw more concrete conclusions concerning how these factors interact with the presence of JOLs and the salience of different cues.

This study tested the predictions of the cue-processing account of reactivity. The results support the idea that reactivity is driven by the enhanced processing of salient cues, which, in the case of uninformative cues and restricted study time, may lead to impaired recall performance. These results contribute to our theoretical understanding of reactivity, but also present a problem for researchers who utilise JOLs as a measure of metacognition. This is because such ratings are not only reactive, but may be reactive in a way that interacts with the presence or absence of particular cues. Using JOLs in experimental designs where a salient cue is present in one condition but absent in another is therefore particularly problematic as any group differences in recall may be an artefact of providing a JOL in such conditions. Future work is needed to clarify the extent to which the inclusion of JOLs in experimental paradigms may inadvertently be contributing to observed group differences.

## Data Availability

Materials and Data are available on the Open Science Framework (https://osf.io/grv3u).
